# A beginner's guide to atomic force microscopy probing for cell mechanics

**DOI:** 10.1002/jemt.22776

**Published:** 2016-09-27

**Authors:** Núria Gavara

**Affiliations:** ^1^ School of Engineering and Materials ScienceQueen Mary University of LondonMile End RoadLondonE1 3NSUK

**Keywords:** atomic force microscopy, cell mechanics, mechanobiology

## Abstract

Atomic Force microscopy (AFM) is becoming a prevalent tool in cell biology and biomedical studies, especially those focusing on the mechanical properties of cells and tissues. The newest generation of bio‐AFMs combine ease of use and seamless integration with live‐cell epifluorescence or more advanced optical microscopies. As a unique feature with respect to other bionanotools, AFM provides nanometer‐resolution maps for cell topography, stiffness, viscoelasticity, and adhesion, often overlaid with matching optical images of the probed cells. This review is intended for those about to embark in the use of bio‐AFMs, and aims to assist them in designing an experiment to measure the mechanical properties of adherent cells. In addition to describing the main steps in a typical cell mechanics protocol and explaining how data is analysed, this review will also discuss some of the relevant contact mechanics models available and how they have been used to characterize specific features of cellular and biological samples. *Microsc. Res. Tech. 80:75–84, 2017*. © 2016 Wiley Periodicals, Inc.

## 1 Introduction

The study of cell mechanics has attracted blooming interest from the cell biology and biomedical communities in the last decade. The mechanical properties of cells affect important factors of cellular function, including shape, motility, differentiation, division, and adhesion to its surrounding extracellular matrix (Moeendarbary & Harris, 2014). As such, cell and tissue stiffness are increasingly regarded as an additional feature of normal and diseased cellular states, being a useful parameter in the study of disease pathophysiology, the development of novel diagnostics, and the advancement of drug discovery (Jin et al., 2010; Kai, Laklai, & Weaver, 2016; Khairallah et al., 2012; Lekka et al., 2012). While there are several established methods to characterize cell mechanics [as reviewed in (Moeendarbary & Harris, 2014) and (Rodriguez, McGarry, & Sniadecki, 2013)], Atomic Force Microscopy (AFM) is probably poised to make the biggest contribution to cell biology in the next decade. Beyond producing nanometre‐scale images of a cell's surface in living physiological conditions and with no sample processing, AFM also provides high‐resolution maps of the cell mechanical properties, thus acting as a reliable indicator of the structure and function of the underlying cytoskeleton and cell organelles.

The increased interest in AFM‐based cell mechanics has paralleled increased efforts by companies manufacturing AFM toward the development of specialized setups for cell biology research. In particular, the newest generation of bio‐AFMs feature seamless integration with epifluorescence (and more advanced) microscopes, larger scanning range in *x*‐*y*‐*z* directions, temperature‐controlled fluid cells to guarantee the long‐term survival of the probed samples and predefined easy‐to‐use measurement protocols and analysis routines. Nowadays, bio‐AFMs are increasingly bought rather than built and are becoming more prevalent in multiuser core facilities. The current ease of use and access, and the multiplicity of choices in AFM operational modes raise a particular risk. In brief, the scientific relevance of any AFM‐based study is as good (or as poor) as the alignment between (1) the research question, (2) the measurement protocol chosen, (3) the assumptions made about the studied sample, and (4) the contact mechanics model used to analyse the raw data. Accordingly, this review is intended for those about to embark in the use of AFM for cell mechanics, and aims to shed some light on the existing choices for probing protocols and data analysis methods that they may face. The first part will briefly summarize the key elements of an AFM, as well as its working principles. The second part will highlight the key choices in designing an AFM‐based experiment to study the mechanics of adherent cells. The third part will describe the main steps in a typical protocol and explain how data is analysed, presenting some of the relevant contact mechanics models used for that goal.

## 2 Fundamentals of AFM

### 2.1 Key elements and operating principles of current AFMs

**Figure 1:**
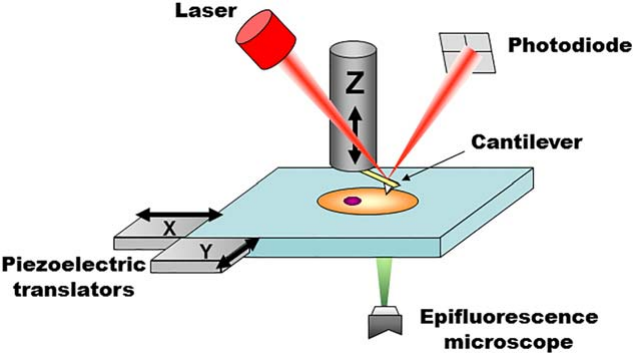
Main elements of a bio‐AFM setup. The tip interacts with the probed sample and attractive/repulsive forces cause the cantilever to bend. Bending is monitored by shining a laser light onto the gold‐coated backside of the cantilever and measuring the position of its reflected light using a four quadrant photodiode. A set of three piezoelectric positioners allows nanometer‐scale movement of the tip with respect to the sample. The stage is typically moved in the *x*‐*y* axis, while the cantilever is moved on the *z* axis. Other configurations are also commercially available, for example, *x*‐*y*‐*z* piezoelectrics moving only the cantilever or only the sample. In commercial systems, the AFM stage is fitted directly onto the body of the epifluorescence microscope (replacing its own stage), to allow seamless integration and an unobstructed optical path for imaging.

AFM was invented in 1986, as one of several scanning probe microscopy (SPM) techniques developed during that decade (scanning tunnelling microscopy being the first one in 1981). As a common theme, all SPM techniques aim at obtaining the topography of a sample with nanometre resolution, by detecting a highly localized interaction between a sharp probe and the sample's surface. The possibility to obtain high‐precision maps is afforded by the use of piezoelectric positioners (typically one for each *x*‐*y*‐*z* dimension) that can move the probe with respect to the sample at subnanometer precision (Figure 1). The topographical maps are obtained in a raster scanning fashion, where each pixel in the map is acquired sequentially, first acquiring all the pixels along a row and then proceeding to the following row. Currently, the use of computers allows reconstructing the topographical image in real time. The commonalities end here, as every SPM technique is based on a different type of probe‐sample interaction, and accordingly features slightly different probes and very distinct methods to quantitatively measure said interaction.

AFM is based on measuring the attractive and repulsive forces acting between the atoms of a sharp tip and those of the sample's surface. The size of the tip determines the lateral resolution of AFM. Accordingly AFM tips designed specifically for imaging have tip radii of less than 10 nm. The tip is attached to a very flexible cantilever, which bends toward or away from the sample when attractive or repulsive forces are present, respectively. Cantilevers are microscopic (tens to hundreds of micrometres length and width) and are etched at the side of a silicon or silicon nitride chips. The chip, which is macroscopic, can be firmly attached to a piezoelectric positioner, which allows ultra‐precise positioning of the cantilever in the vertical direction (*z*‐axis). Importantly, force‐induced cantilever bending and piezoelectric‐based cantilever movement take place in roughly the same vertical axis, which is perpendicular to the surface of the sample.

**Figure 2:**
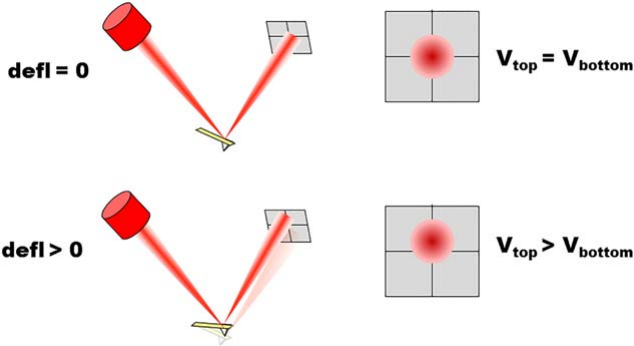
Optical‐based detection of cantilever deflection. *(Top)* When the cantilever is resting undisturbed, the photodiode is manually placed in such a way that half of the laser spot reaches the top quadrants, and the other half the bottom quadrants. *(Bottom)* When the cantilever deflects, the laser spot reaches the photodiode at a slightly different location, causing the output voltages for the top and bottom quadrants to be different.

In most AFMs, the bending of the cantilever (typically referred to as deflection) is detected by optical means. In particular, a laser light is reflected from the cantilever and detected by a quadrant photodiode (Figure 1). While the cantilever is undeflected (usually when resting far away from the sample), the photodiode is manually positioned in such a way that half of the laser spot reaches the top quadrants, and the other half the bottom quadrants (Figure 2). When properly adjusted, the difference between the photovoltage output by the top and the bottom quadrants ( $\Delta V=V_{top}-V_{bottom})$ is zero. When the cantilever interacts with the sample and bends, the laser light is reflected at a slightly different angle, changing the way the laser spot reaches each quadrant and thus the value of Δ*V*. Of note, Δ*V* is proportional to cantilever deflection (in the small deflections regime), and its sign reveals whether bending is caused by attractive or repulsive forces. By measuring Δ*V*, the system monitors the deflection of the cantilever in real‐time (<.1 ms readout time) and with high precision (<.01 nm accuracy) (Butt, Cappella, & Kappl, 2005).

### 2.2 AFM for high‐resolution topography imaging

To obtain a topographical image using AFM, the tip is brought to contact or near‐contact with the surface of interest and it is raster scanned over it. The AFM system continuously monitors the deflection of the cantilever and then adjusts in real‐time the vertical position of the cantilever with respect to the sample, to keep the deflection constant. By means of this feedback mechanism, the tip of the cantilever is kept at a constant distance from the sample as it “glides” over it. Conversely to what one may assume, it is the information encoded in the vertical position of the cantilever, rather than its deflection, what is used to reconstruct the topography of a sample. Being used as feedback parameter, the deflection of the cantilever remains fairly constant on a finely tuned scanning measurement. What has been described so far is widely referred to as “contact mode” imaging. A complementary approach is “tapping mode,” in which the cantilever is forced to oscillate near its resonance frequency. When the tip is brought near the sample, the cantilever's resonance frequency changes slightly, due to increased forces acting between the tip and the sample. This effect is observed indirectly, by monitoring changes in the amplitude of oscillation at a fixed frequency near the resonance peak. The amplitude of oscillation depends on the distance between the tip and the sample, thus being a useful feedback parameter to adjust cantilever height as the topography of the sample changes. In brief, the parameter “amplitude” in tapping mode is the counterpart to the parameter “deflection” in contact mode, each being used to drive the feedback loop that adjusts cantilever height positioning during raster scanning. In tapping mode the interaction between the tip and the sample is reduced (both in duration and amount of force), and is thus preferred as a gentler approach to image biological samples. All AFM imaging modes are based on raster scanning, and thus the time required to acquire an image scales with the number of pixels used. Unfortunately, AFM imaging is not a particularly fast technique. While subsecond acquisition times have been achieved for particular samples (mostly isolated proteins or small flat areas of a cell (Colom, Casuso, Rico, & Scheuring, 2013; Kodera, Yamamoto, Ishikawa, & Ando, 2010; Vielmuth, Hartlieb, Kugelmann, Waschke, & Spindler, 2015; Yoshida et al., 2015), a good‐resolution image of a whole adherent cell's surface will require at least tens of seconds of acquisition time.

### 2.3 Force measurements for the mechanical characterization of biological samples

**Figure 3:**
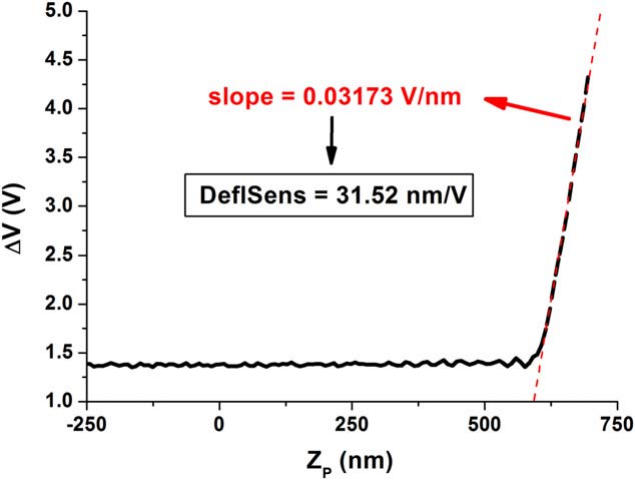
Calibration of the deflection sensitivity. The cantilever is ramped over a very stiff (unindentable) surface. Accordingly, all downward piezoelectric motion will be equal to cantilever bending (right part of the graph). The inverse of the measured slope (red line) corresponds to the sought deflection sensitivity (in nm/V).

While obtaining high‐resolution topography of cellular surfaces is a useful feature, the key advantage of AFM for cell mechanics is the possibility to perform force measurements at desired cellular locations using the tip of the cantilever as indenter. Precise force measurements are possible because the cantilever behaves as a hookean spring, whose stiffness (*k_c_*) can be readily determined. As explained above, AFM measures with high precision the deflection (*d*) of the cantilever, and thus forces (*F*) acting on the cantilever tip are easily computed as $F=k_{c}d$. AFM‐based mechanical characterization is based on using the AFM's tip to apply force onto the sample, while tracking how the sample deforms in response to said force. During the measurement, the vertical displacement of the cantilever and its deflection are recorded simultaneously, and later converted to force‐versus‐displacement curves, briefly called force curves. To obtain a force curve, the cantilever is moved toward the sample in the normal direction. As illustrated in Figure 3, this first part appears as a flat line in the force curve, because the tip is still too far away from the sample to experience any interaction force. Then, depending on the probed sample and the working conditions, the tip may experience observable attractive forces when it is in near proximity to the surface. The tip is considered to be “in contact” with the sample when repulsive forces are first observed (in Figure 3, onset of positive deflections in the force curve). Cantilever movement proceeds until a preset maximum force is reached (furthermost point in force curve), and then the direction of travel is reversed and the cantilever is moved away from the sample. The trigger mode for the maximum force is preferably set to “relative” meaning that the AFM actually tracks the difference between the lowest and highest force values measured in a single force curve. Cantilever motion is reversed when a certain threshold for *F*_max_ *‐F*_min_ is reached, rather than a certain *F*_max_. The process described is typically performed continuously as a loop, with triangular waves used to define the movement of the cantilever. Each individual cycle of cantilever motion is called a ramp, and is divided in an “approach” and “withdraw” parts, according to the direction of motion of the cantilever with respect to the sample. The terminology actually varies among different AFM suppliers or research groups, and the two parts of the ramp may also be referred to as “extend” and “retract.”

## 3 Choosing the Optimal Working Conditions for a Cell Mechanics Experiment

AFM users face a number of choices when defining a measurement protocol, spanning from the stiffness of the cantilever and the shape of the indenting tip to the best location to probe on the cellular surface, the probing frequency or the indenting force/depth. Accordingly, a discussion on those aspects will be provided below, aimed at helping first‐time users to define a measurement protocol that aligns with their research question.

### 3.1 Cantilever tip shape

The choice of AFM tips to perform cell mechanics measurements was a source of heated debate when the first protocols were proposed two decades ago (Radmacher, Fritz, Kacher, Cleveland, & Hansma, 1996). The consensus then was that sharp conical (or pyramidal) tips should be used for cellular imaging, while large radius (∼10 µm diameter) colloidal probes should be used for mechanical characterization (Dimitriadis, Horkay, Maresca, Kachar, & Chadwick, 2002). Since then, studies have shown that cell stiffness can be reliably measured using “sharp” probes and that the values obtained are similar to those found with spherical probes (Chiou, Lin, Tang, Lin, & Yeh, 2013; Rico et al., 2005; Vargas‐Pinto, Gong, Vahabikashi, & Johnson, 2013). Indeed, commercial pyramidal tips are typically blunted (> 100 nm radius), which reduces the actual stress experienced by the probed cell. In this connection, a number of studies have reported no detrimental effects due to persistent probing of an individual cell with a pyramidal tip (Gavara & Chadwick, 2016; Haydon, Lartius, Parpura, & Marchese‐Ragona, 1996). The key advantage to using sharp tips is the possibility to combine simultaneous high‐resolution mapping of the cells' topography with localized mechanical properties and cell adhesion, without having to exchange tips halfway through the experiment. Finally, sharper probes are able to penetrate deeper into the probed sample, given a certain amount of applied force. As a result, a number of studies have focused on mechanical tomography of adherent cells, using large indentations to report on the distinct mechanical contribution of cytoplasmic elements found deep under the cellular cortex (Pogoda et al., 2012; Roduit et al., 2009).

The use of large colloidal probes remains preferable when aiming to understand the cellular mechanical response to whole‐cell stimuli. This is of particular interest in mechanobiology studies, because a larger probe better mimics the type of mechanical stimuli that a cell may experience in its environment (Haase & Pelling, 2015). The aim here is not to catalogue with high resolution the individual mechanical elements of a cell, but rather to measure how these elements respond collectively to a global load. Another reason for favoring the use of spherical probes is the wider availability of contact mechanics models suited for that type of probe. In particular, most models accounting for attractive or long‐range interactions between the sample and the probe are only solved for spherical probes. In these cases, it is unadvisable to perform AFM experiments using a pyramidal/conical tip, if the results will subsequently be analysed with a model appropriate only for spherical tips. Taking that into account, ∼1 µm diameter spherical probes are often used, which enable localized probing in the µm^2^ range in combination with a suitable contact mechanics model (Efremov, Bagrov, Kirpichnikov, & Shaitan, 2015).

Finally, it is worth mentioning some less prevalent tip shapes, such as flat cylindrical indenters or tip‐less cantilevers. Flat indenters are particularly useful when a constant contact area between the probe and the sample is desirable. This is the case in cell‐adhesion studies, in which an estimate of the adhesion force per surface area is sought (Acerbi et al., 2012; Rico, Roca‐Cusachs, Sunyer, Farré, & Navajas, 2007). In the case of tip‐less cantilevers, they have been specially used when probing loosely attached spherical objects, such as nonadherent cells or isolated cell nuclei (Cartagena‐Rivera, Logue, Waterman, & Chadwick, 2016; Chaudhuri, Parekh, Lam, & Fletcher, 2009; Lee et al., 2015; Stewart et al., 2013). In addition, tip‐less cantilevers are a good substrate to attach living cells, which are then used as biological probes to measure adhesion strength of cell–cell interactions (Benoit & Selhuber‐Unkel, 2011; Moreno‐Cencerrado et al., 2016).

### 3.2 Cantilever stiffness

As highlighted above, estimates of cellular elastic modulus are obtained by relating force applied onto the sample with deformation borne by it. Accordingly, an optimal force measurement should display a large amount of cantilever deformation, but also a marked degree of sample indentation. This is achieved by using cantilevers whose stiffness matches that of the probed sample. Cell studies typically use cantilevers stiffnesses ranging between .01 and .6 N/m. Of note, cantilever chips frequently contain an array of 4–6 cantilevers, each with a different stiffness spanning the aforementioned range (Torre, Ricci, & Braga, 2011). Since they are lined‐up closely in the chip, a researcher may easily switch between them during the course of an experiment, by simply moving the laser spot at will. This is particularly useful at the initial stages of a study, to find the cantilever stiffness that best matches the stiffness of the probed sample.

While the predicted stiffness of the sample should serve as a good initial guide toward choosing the stiffness of the cantilever, there are also reasons favoring the choice of slightly stiffer cantilevers. The adhesiveness of the sample is probably the most critical one, as too soft cantilevers may remain bound to very adhesive samples for the whole of the withdraw curve. If the cantilever does not detach from the sample at the end of the ramp cycle, the following approach curve will not have a flat noncontact part region. Rather, large negative deflections will be measured when the cantilever is furthest away from the sample, and will render future offline analysis futile. It is possible to solve this issue by using very long ramps, which bring the cantilever far away from the sample so that detachment can take place. Nevertheless, this comes at the expense of higher tip velocities (for a given probing frequency), which may give rise to hydrodynamic contributions of the liquid environment surrounding the cantilever (Alcaraz et al., 2002). In addition, as the number of data points recorded per force curve is usually limited to 1,024, larger noncontact regions reduce the number of “useful” data points corresponding to the contact region, which are the only ones later fitted to obtain an estimate of elastic modulus.

A second reason to use stiffer cantilevers is the fact that their resonance frequency is also higher. The resonance frequency imposes limits of operation for experiments that involve ramping the cantilever at high velocities. This has proven crucial in the newest generation of AFMs, which perform force curves at the kHz regime (Smolyakov, Formosa‐Dague, Severac, Duval, & Dague, 2016). In particular, the resonance frequency of the cantilever should be much larger than the probing frequency, otherwise the results obtained will be a combination of the viscoelastic response of the probed sample and the probing cantilever.

### 3.3 Probing depth, frequency, and cellular location

Unlike tip shape and cantilever stiffness discussed before, parameters such as indentation depth, location and probing frequency are readily changed during the course of an experiment, via software. That being said, it is important to carefully establish an optimal range of operating values, not only to best address a given research question but also to compare one's results with those obtained by other researchers.

Cells are not a simple fluid‐filled structure, but rather contain distinct intracellular structures that may display distinct mechanical properties(Moeendarbary & Harris, 2014). Accordingly, decisions on the indentation depth and the cell location to probe should be based on the particular intracellular structure of interest. For example, a number of studies have focused on the mechanical properties of either the actin cortex or the underlying stress fibers, using indentation depths <400 nm or > 1 µm, respectively (Gavara & Chadwick, 2016; Vargas‐Pinto et al., 2013). Furthermore, studies aimed at the mechanical tomography of adherent cells use very large indentations, but later dissect out the mechanical contribution of different intracellular structures according to their location along the cell depth (Pogoda et al., 2012; Roduit et al., 2012, 2009). On a different note, studies focusing on, for example, the nucleus or lamellipodia typically use optical images of the cell of interest to position the cantilever tip above the desired intracellular structure before probing.

AFM users have been often cautioned against probing thin areas on the cell periphery, or using indentations larger than 20% of the cell height. Indeed, in both situations, the presence of the stiff glass substrate may result in artifactually large stiffness values. Nevertheless, rather than being overly conservative in the range of indentations and cell locations to probe, it is more advantageous to use a contact mechanics model that takes into account the mechanical contribution of the stiff substrate. Such models exist both for spherical and conical tips and are easily used on routine force curves (Dimitriadis et al., 2002; Gavara & Chadwick, 2012).

Cells are viscoelastic and as a result the measured elastic moduli will depend on the frequency at which the cantilever is ramped. The dependency of elastic modulus on probing frequency follows a weak power law with exponents ranging from .10 to .25 (Alcaraz et al., 2003; Hecht et al., 2015; Rother, Nöding, Mey, & Janshoff, 2014). While in the past this has not been a marked issue, the newest AFMs can obtain force curves using a much wider range of ramping frequencies, up to 2kHz. It is therefore important to take frequency‐dependent effects into account when attempting to compare newly published data obtained with very high ramping frequencies versus years‐old published data obtained at <1 Hz ramping frequencies.

## 4 Calibration Routine Force Measurement Experiments

The underlying principle of AFM‐based cell mechanics is indeed simple: a known force is applied onto the sample and its resulting deformation is measured. Then, by relating the two, the stiffness of the sample can be estimated. The measurement protocol is nevertheless often difficult to conceptualize for a first‐time user simply due to the kind of raw data that is actually obtained in an experiment. As explained above, force experiments are based on obtaining force‐displacement curves. Nevertheless, “force” is not directly measured, and actually, neither is cantilever deflection. Truly, the raw data here corresponds to the difference in photovoltage between the quadrants of the photodetector, previously introduced as Δ*V*. Things are not straightforward either in the case of “displacement.” Here, the displacement that is truly measured is that of the piezoelectric positioner, to which the cantilever chip is firmly coupled to (typically referred to as *Z_p_*). In fact, raw data is the length of the piezoelectric positioner. Together, a force curve in its “rawest” form has the shape and units displayed in Figure 3. It is fascinating that even though AFM is based on tip‐sample interactions taking place in a volume smaller than 1 µm^3^, the raw data is generated by macroscopic components that are centimetres away from the probed volume. As a result, an AFM force experiment requires a series of calibrations before the sample of interest can be probed, as well as postprocessing of the raw data, usually done offline. Commercial systems increasingly incorporate predefined routines to guide the user through the calibration steps and the offline analysis.

### 4.1 Calibration of the deflection sensitivity

The centre‐piece of any AFM experiment is the behavior of the cantilever, specifically changes in its deflection (or amplitude of oscillation). Accordingly, the first step in an AFM experiment is always to calibrate the signal output by the photodiode, so that it can be translated to cantilever bending. The total reflected light reaching the photodiode may depend on a number of things, including the transparency of the liquid buffer or any other components the laser travels through (e.g., the optical path of the cantilever holder) or the reflectivity of the gold layer coating the backside of the cantilever. Furthermore, the value of Δ*V* for a given cantilever deflection will depend on the specific location where the laser spot impacts on the cantilever. Deflection sensitivity may change slightly during the course of an AFM session, and it is recommended to recalibrate it often, for example, when switching samples or even before each cell is probed. The procedure to calibrate deflection sensitivity is simple but elegant, and it only requires ramping the cantilever against a very stiff surface (typically a bare region of glass anywhere in the coverslip containing the adherent cells to be studied). Figure 3 shows one such example. In the rightmost flat part of the curve, the tip has not still reached the sample, and thus remains undeflected (no changes of Δ*V* in *y*‐axis). Once the tip of the cantilever reaches the glass surface, the additional downwards movement of the *Z* piezoelectric will be equal to cantilever bending, as illustrated by the linear slope found in the rightmost part of the curve. Accordingly, the slope of the linear part will be the inverse of the calibration factor needed. Commercial systems already incorporate preset routines to perform this calibration, and usually switch to presenting data as cantilever deflection once the calibration has been carried out.

### 4.2 Calibration of cantilever stiffness

The next step toward obtaining force data involves measuring the stiffness of the cantilever, so that values of deflection can be directly translated to force using Hooke's law. Cantilevers are supplied with information on their nominal stiffness based on their shape and composition (Neumeister & Ducker, 1994), or can even be supplied precalibrated on an individual basis. Nevertheless, it is advisable to measure their stiffness as part of the calibration procedures carried out at the start of an AFM session. While several methods exist [reviewed in (Burnham et al., 2003)], the thermal fluctuations is preferred nowadays because it is quick and can be performed in liquid conditions immediately after calibrating the deflection sensitivity. The thermal fluctuations method is based on the fact that water molecules in the bathing solution are continuously colliding with the cantilever surface, giving rise to very small but random fluctuations of the cantilever bending (typically regarded as noise). By means of the equipartition theorem, one can use the magnitude of the thermal fluctuations to estimate the stiffness of the cantilever (Butt & Jaschke, 1995) (a stiffer cantilever will display smaller‐magnitude thermal fluctuations for a given temperature of the bathing liquid). The specific calibration protocol is built‐in in most commercial AFMs and it is performed with the resting cantilever far away from the sample. First, the thermal fluctuations of the cantilever are recorded for at least 10 s and later converted, via software, into a power spectrum. Typically, the power spectrum displays more than one peak, corresponding to the different resonance modes that the cantilever can engage in. In built‐in routines, the user is prompted to select the first peak (corresponding to the first harmonic oscillation and typically the one displaying the largest peak), and the integral under the peak is then computed. The stiffness of the cantilever is readily estimated by combining the result of the integral with Boltzmann constant and the temperature of the liquid buffer (Butt & Jaschke, 1995).

## 5 Acquisition of Force Curves and Data Processing

### 5.1 Acquisition of force curves

Once the calibration procedures have been carried out, and assuming that parameters such as ramping frequency and indentation force/depth have already been established during the initial stages of the study, obtaining force curves in adherent cells is a fairly automated and high‐throughput procedure. Typically, an optical or fluorescence image is first recorded and used to direct the cantilever to the areas of interest. Commercial systems then allow the user to define lines or squared/rectangular grids, detailing also the spacing between points in the grid. Some commercial AFM systems (e.g., Nanowizard from JPK) also allow for nonrectangular grid arrays, and most include the option to set manually a list of user‐defined coordinates by clicking on a previously obtained topographical or optical image of the sample. Depending on the total number of cell locations probed and the ramping frequency, the whole procedure may take from seconds to minutes. The user is simply left to monitor the progress of the acquisition, checking, for example, that the cell morphology is not negatively affected by repeated probing, or that no cellular debris become attached to the cantilever tip.

**Figure 4:**
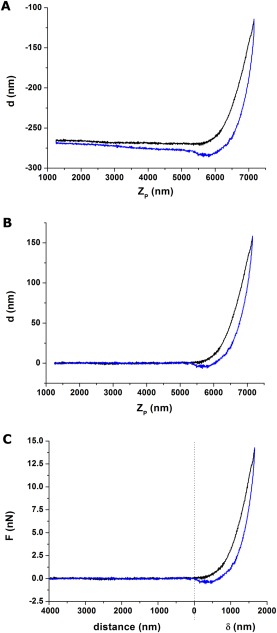
a) Raw data corresponding to a ramp performed using a pyramidal tip at 1Hz. Black line corresponds to approach curve and blue line to withdraw curve. The left part of the curve corresponds to the noncontact region (impacted by a very mild tilt and also hydrodynamics‐induced splitting between the approach and withdraw curves). The right part corresponds to the contact region of the ramp. The nonlinear behavior of the curve suggests that the sample is being indented (increasing contact area between indenter and sample). The lower but positive deflection values observed for the withdraw part suggest that the sample is viscoelastic. b) The noncontact part is used to correct for tilt and hydrodynamics effects. The behavior in the right part of the curve indicating a soft, viscoelastic sample is preserved. c) Once the contact point is established, the force curve can be subdivided into two regions dominated by tip‐sample distance (left part) and sample indentation (right part). Cantilever deflection (nm) has been converted to force (nN) using the known cantilever stiffness (.09 N/m).

Once data acquisition is finalized, the user may choose to use online built‐in methods to obtain mechanical information of the sample, or export the data and analyse it offline using commercial, open‐source (Hermanowicz, Sarna, Burda, & Gabrys, 2014; Roduit et al., 2012) or custom‐built analysis routines (Benitez, Moreno‐Flores, Bolos, & Toca‐Herrera, 2013; Gavara, 2016). Irrespective of the approach chosen, there are some common data analysis steps that all those routines will perform, and they will be described in the following sections. A representative force curve (approach part of the ramp) will be used in the following sections to illustrate the step‐by‐step analysis. Figure 4a shows its initial form, presented as *Z_p_* versus *d*.

Often the approach and withdraw parts of the force curve are not identical. One of the reasons is hydrodynamic drag on the cantilever, most obvious as a splitting of the noncontact parts of the approach and withdraw curves. This offset is proportional to velocity of the cantilever, and can be reduced by either using shorter ramps or slower ramping frequencies. In the contact regime a difference between the approach and withdraw parts is an indication of plastic deformations or most typically, viscoelastic behavior of the sample. In both situations, the force of the withdraw curve is lesser than the force of the approach curve, but it remains positive (repulsive). On the contrary, negative forces on the withdraw curve indicate adhesive forces between the tip and the sample. A careful inspection of the force curves, aimed at identifying any of this features, may inform us on which contact mechanics model should be used to analyse the data.

### 5.2 Data preprocessing using the noncontact part

The leftmost part of the force curve contains no useful information for the computation of mechanical properties, but it is extremely useful in the preprocessing of the data. One would expect the noncontact part to be flat, with deflection values close to 0. That is hardly ever the case, since the location where the laser spot reaches the photodetector tends to drift during the course of an AFM session. As a result, *d* values in the noncontact part, as well as *F*_min_ (introduced in section 2.3 above) is unlikely to be 0, thus justifying the benefits of using a “relative” trigger mode during the acquisition of the force curves. Similarly, minute misalignments between the laser path, the piezoelectric displacement and the coupling of the cantilever chip to the piezoelectric may add a small slope to the whole force curve. To correct these issues, a selected range in the noncontact part is typically fitted to a first order polynomial, and the deflection values predicted from the fit are then subtracted from the measured deflection values, as illustrated in Figure 4b.

### 5.3 Determination of the contact point

The next step is to transform *Z_p_* into values that truly reflect the *relative* position of the tip with respect to the sample. This is achieved by identifying the contact point (CP), that is, the *Z_p_* value at which the tip reaches the sample. Values to the left of the CP will represent “distance” between the tip and the sample, and values to the right of the CP will represent “indentation” of the sample by the tip (Figure 4c). At CP, the value for the *x* axis should thus be 0. If there are no attractive or long‐range interactions between the tip and the sample, *d*(CP) will also be 0. It should be noted that, while the cantilever is moved down into the sample, both cantilever bending and sample deformation take place. Therefore, to compute sample indentation (*δ*), the bending of the cantilever is subtracted from the downwards movement of the cantilever using $\delta =Z_{p}-d-(Z_{p}(CP)-d(CP))$, where *δ* is offset so that at CP, *δ* = 0.

**Figure 5:**
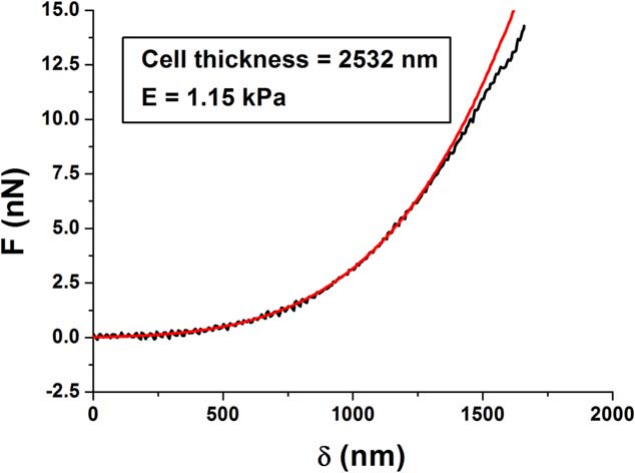
The contact part (approach curve) is fitted with an adequate contact mechanics model. In this case, data has been fitted using BECC model for thin samples (Gavara & Chadwick, 2012), given the fact that indentations correspond to > 50% of cell thickness. The discrepancy between the data and the fit for the largest indentations is likely to reflect the fact that cortical regions are stiffer than regions deep inside the cytoplasm, as shown by (Pogoda et al., 2012; Vargas‐Pinto et al., 2013).

Precise identification of the CP is critical to obtain reliable estimates of cell elastic moduli, since inaccuracies of, for example, 50 nm can give rise to ficefold overestimations or underestimations of the computed cell stiffness (Gavara, 2016; Shoelson, Dimitriadis, Cai, Kachar, & Chadwick, 2004). In addition, given the large number of force curves typically obtained per experiment, CP determination has to be done in a fully automated and moderately fast manner. A number of different strategies have been proposed to identify the CP. The simplest approach is based on a sequential inspection of the force curve, where each point of the curve is assessed as potential CP (Hermanowicz et al., 2014; Shoelson et al., 2004). For each CP candidate, the *Z_p_* versus *d* curve is converted to *δ* versus *F* and then fitted with the chosen contact mechanics model, to obtain an estimate for *r* ^2^ or RMSE. The CP candidate with the highest *r* ^2^ or lowest RMSE is then established as CP. Other strategies have been proposed, typically performing better than the method just described (Benitez et al., 2013; Gavara, 2016). Finally, once CP has been established, the corresponding *δ* versus *F* curve is obtained and fitted with a contact mechanics model, to obtain estimates of elastic moduli or other mechanical parameters (Figure 5).

## 6 Contact Mechanics Models Often Used in Cell Mechanics Studies

The choice of contact mechanics model is mainly based on three criteria: (1) whether there are long‐range or attractive interactions between the tip and the sample, (2) whether the material is linear or nonlinear elastic (that is, whether a material appears to be softer or stiffer, depending on the amount of deformation that it undergoes), and (3) the shape of the tip. A number of models have been developed over more than a century to tackle at least two of these criteria. Unless stated otherwise, all models described below assume a finite indenter interacting in the normal direction with a flat, semifinite, homogenous, isotropic, and purely elastic sample.

The family of Hertzian models deals with situations in which no attractive forces are present between the tip and the sample, that is, only repulsive forces arise when the tip gets in contact and proceeds to indent the sample. Hertzian models have been solved for the principal tip shapes: spherical [Hertz (1881)], conical [Sneddon (1965)], and pyramidal [Bilodeau (1992)]. In addition, further models have been developed for thin samples [(Dimitriadis et al., 2002), (Gavara & Chadwick, 2012)] and blunted tips (Rico et al., 2005), or to account for viscoelasticity effects [(Alcaraz et al., 2003), (Rebelo, de Sousa, Mendes Filho, & Radmacher, 2013)]. Attractive interactions are either studied using the DMT model [stiff samples, small‐radius indenters and small surface energy (Derjaguin, Muller, & Toporov, 1975)] or the JKR model [soft samples, large‐area indenter and large surface energy (Johnson, Kendall, & Roberts, 1971)]. Long‐range interactions due to a polymer brush region over the sample's surface have also been modelled (Iyer, Gaikwad, Subba‐Rao, Woodworth, & Sokolov, 2009). The DMT, JKR, and polymer brush models are only solved for a spherical indenter over a flat surface, thus limiting the choice of tips to colloidal probes. All models presented so far assume the sample to be linear elastic. For nonlinear elastic materials, Fung's (Fung, Fronek, & Patitucci, 1979) or Ogden's (1972) hyperelastic models are typically used, again solved only for spherical indenters. Irrespective of the model used, certain parameters are assumed to be known (e.g., Poisson's ratio, which is often set to .5, or the radius/opening angle of the spherical/conical tip). The unknown parameters that are obtained by fitting *δ* versus *F* (or *distance* vs. *F*) curves are the Young's modulus (*E*), and when suitable the surface energy γ or the viscosity μ. Typically, the DMT and JKR models are fitted to the withdraw curve of the ramp, while Hertzian models tend to perform best for the approach part of the ramp. Models accounting for viscoelasticity are based on comparing the approach and withdraw curves.

A situation may arise in which a sample is, for example, both hyperelastic and displays strong adhesive forces. Unfortunately, there are no salomonic solutions available, at least using an analytical solution. One is thus forced to prioritize the material behavior that is of highest interest choose a model, acknowledging the fact that the assumptions made by the model are not fully satisfied in the probed sample. Given their mechanical features and morphology, adherent cells and biological tissues are particularly ill‐poised to fulfil all the assumptions of any contact mechanics model described so far. In particular, cells have a limited thickness and in some areas display large changes in height. Furthermore, they are not homogeneous and the often parallel organization of their actin stress fibers (Gavara & Chadwick, 2016; Roca‐Cusachs et al., 2008) suggest that they are also not isotropic. That being said, the models described above have been successfully used to study cell mechanics, focusing on, for example, how the organization of the cytoskeleton modulates the elastic moduli of the cells (Gavara & Chadwick, 2016; Roca‐Cusachs et al., 2008), how inflammatory mediators change the hyperelastic properties of endothelial cells (Kang et al., 2008), or how malignancy affects the viscoelasticity of cells (Rebelo et al., 2013; Rother et al., 2014), or the length of their glycocalyx (Iyer et al., 2009).

## 7 Conclusions

Over the last five year, AFM has overcome the main limitations preventing it from being a widespread technique for basic science and biomedical research. Current AFMs allow mechanical characterization of adherent cells in a fast, high‐throughput and single‐cell manner. Furthermore, the obtained high‐resolution maps for cell topography, stiffness and adhesion are readily overlaid with matching fluorescence or even super‐resolution images of the probed cells. Fully quantitative mechanical characterization of cells remains somehow limited due to the complex nature of cells, which prevents their complete description using a single contact mechanics models. The use of finite element models can be advantageous to that end, even though its implementation may be limited to highly trained researchers. Finally, with the increased relevance of mechanobiology, the value of AFM is slowly extending beyond being a passive method to characterize cells, toward serving also as a tool to actively elicit mechanosensitive responses in cultured cells.
